# Towards Superhuman Imitation Learning for Sequential Head-and-Neck Cancer Treatment Decisions

**DOI:** 10.1145/3748522.3779849

**Published:** 2026-06-09

**Authors:** Filippo Corna, Xinhua Zhang, Guadalupe Canahuate, Serageldin Attia, Abdallah Mohamed, Mohamed Naser, Clifton Fuller, Elisabeta Marai

**Affiliations:** 1 Department of Computer Science, University Illinois Chicago, Chicago, Illinois, USA; 2 Department of Computer Science and Engineering Politecnico di Milano, Italy; 3 Department of Electrical and Computer Engineering, University Of Iowa, Iowa, USA; 4 Department of Radiation Oncology, The University of Texas MD Anderson Cancer Center, Texas, USA

**Keywords:** Healthcare AI, Imitation Learning, Reinforcement Learning, Subdominance Loss, Head and Neck Cancer

## Abstract

This work presents the design of a simulator-driven imitation learning approach for sequential treatment decisions in head and neck cancer, built around Superhuman Policy Gradient Optimization (SPGO). Rather than simply replicating physicians’ actions, the method leverages a clinical simulator to generate complete patient trajectories and incorporates an inverse-reinforcement-learning–inspired loss that rewards policies for outperforming experts on key clinical outcomes, including relapse rates and long-term toxicities.

## 1 Introduction

Head and Neck Cancer (HNC) includes malignant tumors arising in the oral cavity, pharynx, and larynx, and represents approximately 3–4% of all cancers worldwide. Its management is inherently complex, involving multi-stage therapeutic decisions that must balance tumor control with long-term functional and toxicity outcomes.

Early software tools for cancer care were primarily used for monitoring patients, but with the advent of modern AI methods they have evolved into genuine decision-support systems capable of guiding oncological treatment planning. Traditional supervised learning approaches, such as behavior cloning, suffer from error compounding and cannot reliably manage sequential decisions. This limitation is particularly relevant in medicine, where identical clinical decisions can lead to different outcomes because patients respond heterogeneously to the same therapy. For this reason, evaluating a treatment policy requires focusing on the final outcomes of the therapeutic trajectory rather than on single-step prediction accuracy.

Since the transition dynamics between clinical states are unknown and we cannot compute gradients through them, classical backpropagation cannot be applied. To address this, we rely on policy gradient methods, which allow us to directly optimize the policy based on outcome-driven signals without requiring explicit transition probabilities.

## 2 Dataset and Simulator

Our study uses a de-identified clinical dataset from the MD Anderson Cancer Center (MDACC) comprising 676 patients diagnosed with head and neck cancer (HNC) and treated between 2010 and 2021. Each patient record integrates:

- **Pretreatment features:** demographics (age, sex, smoking), tumor site and stage, HPV status.
- **Treatment decisions:** three sequential treatment stages including definitive surgery (DS), induction chemotherapy (IC), and radiotherapy (RT) with or without concurrent chemotherapy (RT/CC).
- **Patient-reported outcomes (PRO):** 22 symptoms measured longitudinally (Baseline, End of RT, Week 6, Month 3, Month 12) covering pain, fatigue, dry mouth, swallowing, and taste difficulties.
- **Treatment Plan Outcomes (TPO):** relapse at year 3, margins, extranodal extension, radiation dose and fractionation.

To realistically model patient evolution under different *given* treatment policies, we leverage a hybrid simulator that combines generative and predictive components [2]. The simulator reproduces the longitudinal clinical dynamics observed in the MDACC dataset, enabling safe experimentation with learned policies in a controlled, reproducible environment. Additionally, the simulator handles missing values in the dataset, providing complete and consistent patient trajectories for policy training and evaluation.

## 3 Methodology

We train three distinct policy networks corresponding to the sequential medical decisions across the treatment pathway. Each network conditions its decision on all prior patient information, capturing the causal and temporal dependencies of the clinical process. The full simulated trajectory of state $s_{t}$ and $a_{t}$, namely

$$\xi =\left(s_{1},a_{1},s_{2},a_{2},s_{3},a_{3},s_{4}\right),$$

encodes the entire decision-making sequence and resulting patient features.

To compare agent and expert trajectories, we employ the *subdominance loss* from **Superhuman Policy Gradient Optimization (SPGO)** [3], which evaluates trajectories across multiple outcome features. Let $f_{k}(\xi )$ denote the value of feature k for trajectory ξ, and $\tilde{\xi }$ be the corresponding expert trajectory. The subdominance loss for feature k can be defined in two ways:

(1) $$\text{Absolute}\;\text{form:}\;L_{k}^{\text{abs}}(\xi ,\tilde{\xi })≔{\left[\alpha_{k}\left(f_{k}(\xi )-f_{k}(\tilde{\xi })\right)+\beta_{k}\right]}_{+},$$

(2) $$\text{Relative}\;\text{form:}\;L_{k}^{\text{rel}}\left(\xi ,\tilde{\xi }\right)≔{\left[\alpha_{k}\left(\frac{f_{k}\left(\xi \right)}{f_{k}\left(\tilde{\xi }\right)}-1\right)+\beta_{k}\right]}_{+},$$

where $[x{]}_{+}=\text{max}\{x,0\},\beta_{k}$ is a target margin, and $\alpha_{k}$ is an adaptive slope updated online. Per-feature losses can be aggregated via:

**Summation (promotes global improvement):**

(3) $$L_{\text{sum}}^{\text{abs}/\text{rel}}(\xi ,\tilde{\xi })≔\sum_{k}L_{k}^{\text{abs}/\text{rel}}(\xi ,\tilde{\xi }),$$

**Maximum (focuses on the worst violation):**

(4) $$L_{\text{max}}^{\text{abs}/\text{rel}}(\xi ,\tilde{\xi })≔\underset{k}{\text{max}}\;L_{k}^{\text{abs}/\text{rel}}(\xi ,\tilde{\xi }).$$

Given a set of expert trajectories $\tilde{\Xi }$, the stochastic reward for a generated trajectory is defined as:

(5) $$R\left(\xi \right)=-L\left(\xi ,\tilde{\Xi }\right)=-\frac{1}{\left|\tilde{\Xi }\right|}\sum_{\tilde{\xi }\in \tilde{\Xi }}L\left(\xi ,\tilde{\xi }\right),$$

where L can be any of the four combinations: absolute/relative × sum/max. Adaptive coefficients $\alpha_{k}$ allow the policy to focus training on the most challenging features [3].

**Training.** The policy networks are trained using the REINFORCE algorithm [1], where the negative subdominance loss serves as the reward. Training proceeds in mini-batches of patients, and gradients are accumulated across all trajectories in the batch. Gradient clipping and the Adam optimizer are applied to ensure stable convergence. This procedure enables the policy to learn across multiple outcome features without relying on predefined scalar rewards, adapting dynamically to optimize challenging objectives.

## 4 Experimental Results

We evaluated six configurations of the subdominance loss (absolute vs. relative differences; sum vs. max aggregation; update-all α vs. max-only α) as described in Section 3. Performance is measured in terms of policy *dominance* over physician decisions using both aggregated toxicity (sum of PROs and TPOs) and majority-of-features metrics.

Across configurations, SPGO achieves dominance in the majority of cases: 61–74% for total toxicity, with the highest rate (73.7%) using relative differences with sum aggregation, and ~70% dominance by majority of features. Absolute or max-only configurations perform slightly worse, reflecting a trade-off between stability and optimization.

**Best configuration:** relative differences + sum aggregation + per-feature α updates; all subsequent results use this configuration.

### 4.1 Comparison with Physician

SPGO policies were evaluated against expert trajectories. Acute toxicity at treatment end is similar to the physician, but SPGO achieves lower toxicity in later stages, indicating improved long-term control without sacrificing immediate treatment effectiveness. When analyzing by symptom type (Fatigue, Drymouth, Swallow, Taste), SPGO aligns closely with physician decision overall but shows reductions in swallowing and taste toxicities, reflecting better handling of lateonset side effects. These results support SPGO’s ability to surpass pure imitation by improving clinically relevant endpoints. Moreover, SPGO reproduces the physician’s relapse pattern with **85.4%** overall agreement. In 5.84% of cases SPGO underperforms (relapse occurs under SPGO but not under physician), whereas in 8.76% of cases SPGO prevents relapse where the physician did not, yielding a **net improvement of +2.92%** of patients. This demonstrates that SPGO maintains clinical plausibility while achieving marked gains in relapse control.

### 4.2 Comparison with Behavioral Cloning

To further compare with imitation-based learning, we trained a standard *Behavioral Cloning* (BC) model. Each BC network mirrors the architecture of the corresponding SPGO policy (three-layer MLP with ReLU activations and dropout) but is trained in a purely supervised manner between predicted and expert actions.

SPGO consistently achieves lower simulated toxicity values compared to the BC policy, demonstrating that the policy-gradient mechanism guided by the subdominance loss enables targeted improvement rather than simple replication of expert behavior. Reductions are especially notable in later timepoints and for swallowing-related symptoms.

On relapse predictions, SPGO and BC agree in 83.95% of patients (46.72% no-relapse, 37.23% relapse). In 6.57% of cases, SPGO underperforms (relapse occurs under SPGO but not BC), whereas in 9.49% of cases, SPGO prevents relapse where BC does not, yielding a **net improvement of +2.92%** of patients. This confirms that SPGO outperforms BC in relapse control under the simulator dynamics.

Comparing individual toxicity outcomes between BC and SPGO shows distributions centered near zero with a slight positive bias, indicating consistently lower toxicity values for SPGO. This effect is most pronounced at later stages and for swallowing-related metrics, suggesting that SPGO improves long-term outcomes through effective optimization rather than stochastic variation.

The full results and paper are available at on MedRxiv, with the same title and author list.

## CCS Concepts

**• Applied computing → Health informatics; • Computing methodologies →** *Reinforcement learning; Learning from demonstrations*.
